# pH-Responsive Hydroxypropyl Cellulose-Based Membranes for Controlled Mass Transport and Drug Release

**DOI:** 10.3390/membranes16060187

**Published:** 2026-05-31

**Authors:** Ahmed Mahmoud Ismail, Ayesha Sattar, Muhammad Amin, Muhammad Asif Shabbir, Mustansar Mubeen, Muhammad Umer, Yasir Iftikhar, Ramy S. Yehia, Basem M. Abdallah, Enas M. Ali, Biju Vadakkemukadiyil Chellappan, Khaled M. A. Ramadan

**Affiliations:** 1 Pests and Plant Diseases Unit, College of Agricultural and Food Sciences, King Faisal University, Al-Ahsa 31982, Saudi Arabia; amismail@kfu.edu.sa; 2 Department of Chemistry, University of Lahore, Sargodha Campus, Sargodha 40100, Pakistan; ayeshasattar140@gmail.com (A.S.); muhammad.amin@chem.uol.edu.pk (M.A.); 3 Department of Plant Pathology, College of Agriculture, University of Sargodha, Sargodha 40100, Pakistan; mustansar01@yahoo.com; 4 School of Breeding and Multiplication (Sanya Institute of Breeding and Multiplication), College of Tropical Agriculture and Forestry, Hainan University, Sanya 572025, China; umer.hzau@outlook.com; 5 Department of Plant Pathology, College of Plant Science and Technology, Huazhong Agricultural University, Wuhan 430070, China; 6 Department of Biological Sciences, College of Science, King Faisal University, Al-Ahsa 31982, Saudi Arabia; ryehia@kfu.edu.sa (R.S.Y.); babdallallah@kfu.edu.sa (B.M.A.); eabdelkader@kfu.edu.sa (E.M.A.); bchellappan@kfu.edu.sa (B.V.C.); 7 Central Laboratories, Department of Chemistry, King Faisal University, Al-Ahsa 31982, Saudi Arabia; kramadan@kfu.edu.sa

**Keywords:** hydrogel membranes, stimuli-responsive swelling–deswelling behavior, controlled swelling–deswelling, drug diffusion, hydroxypropyl cellulose

## Abstract

The swelling-regulated transport properties of modified and cross-linked HPC-based hydrogel formulations containing NaCMC and citric acid were studied as stimuli-responsive polymeric membranes under various conditions, including deionized water. Physiological conditions were simulated by evaluating various pH conditions (1.2, 6.8, and 7.4). The pseudo-second-order kinetic model best described the swelling process, suggesting that both solvent uptake capacity and polymer network relaxation contribute to the extent of swelling. The swelling behavior of the hydrogel formulations was significantly influenced by salt concentration. The modified HPC hydrogel system exhibited stimuli-responsive swelling–switching behavior under saline, water/ethanol, and acidic/basic environments, demonstrating reversible swelling–deswelling cycles. Maximum swelling was observed in water at pH 7.4. In contrast, abrupt deswelling in an ethanol solution at pH 1.2 reduced hydrogel swelling and water uptake. The effect of temperature on the swelling behavior of the hydrogel and its thermo-responsive swelling behavior was also evaluated. Drug release behavior suggested diffusion-mediated release through the swelling hydrogel matrix. These findings suggest that the modified HPC-based hydrogel system may be useful for pH-responsive oral drug delivery applications.

## 1. Introduction

**Figure 1:**
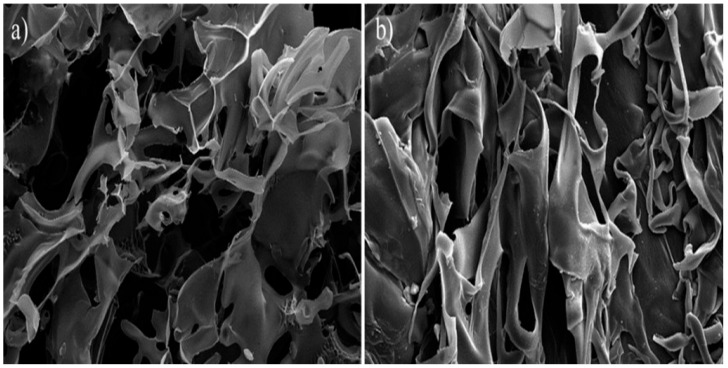
SEM micrographs of HPC-based hydrogels showing porous interconnected structures: (**a**) hydrogel with a more open and porous morphology; (**b**) hydrogel with a comparatively denser and more compact network structure.

Hydrogels are 3D polymer networks that are highly hydrated, capable of absorbing and retaining substantial volumes of biological fluids and liquids. Their unique ability to swell and hold water makes them valuable materials, especially in biomedical, pharmaceutical, and environmental applications. Hydrogels have great biocompatibility, biodegradability, and versatility, which is why they are used in controlled tissue engineering, drug delivery systems, biosensors, and wound dressings [1,2,3]. Hydrogels’ swelling behavior is critical for defining their ability and solubility, as they absorb water and/or other solvents. Osmotic phenomena within hydrogels are well established to induce substantial volumetric expansion, arising from osmotic pressure gradients between the polymer network and the surrounding medium, along with the type of solvent used, the ionic strength of the solvent, temperature, and pH [3,4]. Besides their traditional uses, hydrogels can be considered soft polymeric diffusion matrices, in which the network structure generated by cross-linking serves as a diffusion barrier, and the degree of swelling may influence diffusion-associated behavior. In recent years, stimuli-responsive hydrogel membranes have also attracted attention in membrane science due to their reversible swelling behavior and environmentally responsive diffusion characteristics. Such hydrogel systems have been investigated for nanofiltration and responsive separation applications, including chemically stable selective membrane layers [5]. Implantable hydrogel-based membranes exhibit dynamic, stimulus-responsive swelling and diffusion behavior that cannot be achieved with rigid membranes, thereby making them potentially useful for controlled drug-delivery applications (Figure 1). Hydroxypropyl cellulose (HPC) is a cellulose derivative consisting of a cellulose backbone modified with hydroxypropyl substituent groups, possessing high water affinity, biocompatibility, and versatile performance that make it a suitable material for hydrogel production. Hydroxypropyl cellulose (HPC) is a linear polymer modified by the attachment of hydroxypropyl groups (–OCH_2_CH(OH)CH_3_). This modification significantly improves its solubility in water and polar organic solvents. As a result, HPC is a suitable candidate for hydrogel formation. HPC is non-toxic, biodegradable, and thermally stable. Such characteristics support its application across various biomedical fields [6,7]. In its hydrogel state, HPC can absorb water and swell significantly without dissolution, making it useful for controlled drug release systems and for diffusion-associated transport, including stimuli-responsive drug-delivery applications. As HPC hydrogels are used in oral drug formulations, they allow modulation of drug release rate as a function of environmental conditions [8]. The hydration behavior of modified HPC-based hydrogels containing ionizable components such as NaCMC is strongly pH-dependent. This property is important for their use in controlled drug-delivery systems. Neutral or mildly alkaline levels (6.8–7.4) offer a significantly greater swelling potential than pH 1 and 1.2, both of which are highly acidic. This property may support the design of pH-sensitive hydrogels that may support site-selective drug delivery in the gastrointestinal tract, particularly for intestinal and colon-targeted oral drug delivery applications (e.g., the small intestine), where pH remains relatively neutral. The pH-dependent swelling behavior of the hydrogel may influence swelling-associated molecular transport and delivery under physiological conditions. This makes the modified HPC-based hydrogel system a stimulus-responsive hydrogel, in which ionic strength (NaCl or KCl) and solvent type (water vs. ethanol) can influence its swelling and deswelling [7,8]. Such hydrogels are often referred to as smart or stimuli-responsive hydrogels. They undergo volume changes in response to external stimuli such as pH, temperature, ion concentration, and solvent polarity. As a result, they exhibit stimulus-responsive swelling and diffusion behavior [4,9]. Such on/off switching behavior could be beneficial for drug-delivery applications, as environmental stimuli may activate the drug, enhancing therapeutic efficacy while reducing crystallization. Such materials exhibit reversible swelling and deswelling, making them very effective in controlled drug-delivery systems. Hydrogel swelling kinetics play a key role in controlling drug release rates. This effect is especially important for hydrogels that swell significantly under physiological conditions such as HPC. This behavior can be utilized for continuous and controlled drug delivery over extended periods. Swelling behavior is typically described using second-order kinetics, which are similar to those dependent on solvent uptake capacity and polymer network relaxation. This model provides a greater insight into the swelling kinetics of HPC hydrogels, particularly in drug delivery applications [9,10]. Apart from swelling kinetics, the stimuli-responsive features of HPC hydrogel are of great interest. It has been reported that modified HPC-based hydrogels containing ionizable functional groups undergo reversible swelling–deswelling cycles in response to changes in pH or solvent conditions. For instance, the hydrogel is maximally swollen at a neutral buffer condition (approximately 7.4), but shrinks remarkably in acidic solutions (pH 1.2). Such behavior is particularly important for oral drug delivery. These properties of hydrogel can be used to control drug release in different sections of the gastrointestinal (GI) tract [9]. Although HPC-based hydrogels have been widely studied for swelling and drug release applications, limited studies have interpreted these systems from the perspective of stimuli-responsive swelling and diffusion behavior under physiological conditions. Therefore, the present work investigates HPC hydrogels as environmentally responsive systems capable of exhibiting swelling-responsive diffusion behavior. Environmental factors such as temperature and salt concentration also influence the swelling properties of HPC hydrogels. These environmental factors also affect ionic strength and the electrostatic interactions between polymer chains of hydrogels [11]. In this study, the swelling properties and stimuli-responsive nature of HPC hydrogel were assessed in response to changes in environmental conditions, including pH, solvent type, and ionic strength. This study focuses on interpreting the hydrogel as a responsive hydrogel system characterized by swelling-responsive diffusion behavior. Moreover, the effects of temperature and environmental factors on swelling behavior and drug release characteristics are also examined to evaluate their potential in controlled drug release and targeted drug delivery applications. The present study primarily focuses on interpreting modified HPC-based hydrogels as stimuli-responsive hydrogel systems with environmentally regulated swelling behavior and diffusion-mediated drug release.

## 2. Materials and Methods

### 2.1. Materials

Hydroxypropyl cellulose (HPC), sodium carboxymethyl cellulose (NaCMC), and citric acid (CA) were used to prepare the hydrogel formulations (Table 1). For the HPC-NaCMC-CA formulation, NaCMC was first dissolved in deionized water under continuous stirring for 45 min, followed by the addition of HPC and further stirring for 18 h until a homogeneous mixture was obtained. Citric acid was then added as a crosslinking agent, and stirring was continued for an additional 4 h. For HPC-CA and NaCMC-CA formulations, the respective polymers were dissolved in deionized water under continuous stirring before addition of citric acid. The resulting hydrogel mixtures were transferred into Petri dishes and dried overnight at 60 °C. The dried hydrogel films were removed, ground, sieved through mesh No. 60, and stored in airtight containers for subsequent characterization and swelling studies.

### 2.2. Scanning Electron Microscopy (SEM) Characterization

The surface morphology and porous structure of HPC hydrogels were examined using scanning electron microscopy (SEM). Before imaging, hydrogel samples were dried and mounted on aluminum stubs using conductive adhesive tape. The samples were sputter-coated with a thin layer of gold before SEM analysis to improve conductivity and image quality. Surface morphology and pore distribution were evaluated to examine the structural characteristics of the hydrogel network.

### 2.3. Analysis of TGA

Thermogravimetric analysis (TGA) of the samples was carried out using an SDT Q650 simultaneous thermal analyzer (TA Instruments, New Castle, DE, USA). Approximately 5–10 mg of each dried hydrogel sample was used for thermogravimetric analysis. The samples were heated from room temperature to 600 °C at a heating rate of 10 °C/min under a nitrogen atmosphere maintained at a flow rate of 90 mL/min.

### 2.4. Characterization of HPC Hydrogel / Physical Properties

The powdered hydrogel was evaluated for its physical properties. These included particle size, angle of repose, tapped density, bulk density, water retention capacity, swelling ability, Hausner ratio, moisture content, and Carr’s index.

### 2.5. Determination of Repose Angle

The flow properties of the hydrogel were evaluated by measuring the angle of repose using the fixed-funnel method described by [12]. In this method, the hydrogel powder was allowed to flow from a stationary funnel onto graph paper, forming a conical pile. The angle of repose (θ) was then calculated via Equation (1).

(1) $$\mathrm{Tan}\theta =\frac{h}{r}$$

where *h* is the height and *r* is the radius of the resulting powder heap.

### 2.6. Density of the Bulk and Tapped Hydrogel

A 1.0 g sample of hydrogel was placed in a graduated cylinder to determine its initial volume (V_b_). The cylinder was then tapped 100 times, and the resulting volume (V_t_) was recorded. These values were used to calculate bulk density (D_b_) and tapped density (D_t_) according to Equations (2) and (3).

(2) $$D_{b}=\frac{Weight\;of\;hydrogel}{Volume\;of\;hydrogel\;\left(V_{b}\right)}$$

(3) $$D_{t}=\frac{Weight\;of\;hydrogel}{Tapped\;volume\;\left(V_{t}\right)}$$

### 2.7. Evaluation of Carr’s Index and Hausner Ratio of Hydrogel

The flow behavior of hydrogels was characterized by measuring the Hausner ratio and Carr’s index [12]. The Hausner ratio is represented by Equation (4):

(4) $$Hausner\;ratio\left(H\right)=\frac{D_{t}}{D_{b}}$$

Carr’s index indicates the extent of particle packing and is expressed as a percentage determined using Equation (5):

(5) $$Carr’s\;index\left(C\right)=100\times \left(1-\frac{D_{b}}{D_{t}}\right)$$

Here, D_b_ represents the bulk density of the hydrogel, while D_t_ corresponds to the tapped density.

#### Moisture Levels of HPC Hydrogel

The hydrogel was weighed before and after drying at 105 °C for 1 h. Moisture content was determined with the help of a Sartorius Thermo Control Infrared Dryer (YTC 01L, Sartorius, Göttingen, Germany).

### 2.8. Capacity of Hydrogel for Water Retention

The hydrogel’s water-retention capacity was determined using a centrifugation-based method. A 1% (*w*/*w*) sample was first prepared in deionized water and centrifuged at 4500 rpm for 30 min under ambient conditions. The liquid phase was carefully decanted, and the hydrated residue was collected and weighed. The sample was subsequently dried at 70 °C until no further weight change was observed, and the final dry mass was recorded. Water retention capacity was expressed as the ratio of the hydrated residue mass to the corresponding dry hydrogel mass [13,14].

### 2.9. Determination of Gel Fraction

To determine the gel fraction, dried hydrogel samples were initially dried in a vacuum oven at 50 °C for 24 h, after which their dry mass (W_0_) was noted. The dried samples were then placed in distilled water and maintained at 37 °C for 24 h to allow swelling and extraction of any soluble fractions. Following this treatment, the hydrogel samples were removed and dried again under the same conditions until a constant weight was reached. The final mass obtained after this step was recorded as W_1_. The gel fraction (GF%) was then determined using Equation (6) [15]:

GF (%) = (W_1_/W_0_) × 100 (6)

All experiments were performed in three replicates to ensure accuracy, and the results are reported as mean values. The experimental data are presented as mean ± standard deviation.

### 2.10. Buffer Solutions Preparation Across pH Range

The swelling response of HPC hydrogels was investigated under different pH conditions using deionized water and buffered media (pH 1.2, 6.8, and 7.4). All buffer systems were prepared fresh before the experiments. The acidic medium (pH 1.2) was obtained by combining potassium chloride and hydrochloric acid solutions (both 0.2 M) and diluting the mixture to a final volume of 1 L with deionized water. For pH 6.8, a phosphate-based buffer was prepared by adjusting a potassium dihydrogen phosphate solution (0.2 M) with sodium hydroxide (0.2 M) and then diluting to the final volume. A similar approach was used at pH 7.4, where potassium dihydrogen phosphate was neutralized with sodium hydroxide and diluted to a final volume of 1 L with deionized water.

### 2.11. Hydrogel Swelling and Deswelling in Water and Ethanol

HPC hydrogel powder (0.5 g) was accurately weighed and placed in a dialysis membrane bag, which was suspended in 50 mL of deionized water in a beaker. All swelling–deswelling experiments were carried out at room temperature (25 ± 2 °C). After some time, the dialysis membrane bag was removed, and excess water was drained. The hydrogel was then allowed to swell for 1 h in deionized water and then placed in ethanol, and the swelling capacity of the HPC hydrogel was calculated via Equation (7):

(7) $$Swelling\;capacity=\frac{W_{t}-W_{0}-W_{c}}{W_{0}}$$

The swelling–deswelling cycle was repeated four times to evaluate the solvent-responsive behavior of the hydrogel.

### 2.12. Reswelling Performance (RSW)

In order to evaluate its reversibility and water reabsorption capacity, the swelling ratio of each hydrogel sample was measured during five subsequent drying–swelling cycles. This process consisted of repeating the swelling, deswelling, and reswelling procedures. After each swelling cycle, the samples were placed in a ventilated oven at 30 °C for 24 h to dry and then analyzed for water reswelling kinetics (RSW). The RSW of the swollen hydrogel was determined by utilizing the standard swelling equation. One drying–swelling sequence was considered a reswelling cycle, and this process was repeated 5 times for each sample.

### 2.13. Swelling Kinetics and Equilibrium Analysis

To evaluate swelling behavior, hydrogel samples (0.5 g) were enclosed in dialysis membrane bags and placed in different media, including deionized water, an acidic solution (pH 1.2), and phosphate buffer solutions (pH 6.8 and 7.4). The pH of each medium was verified using a JENWAY 3510 pH meter (Jenway, London, UK) before the experiment. The samples were maintained under these conditions for 24 h, during which their mass was recorded at selected time intervals. Swelling characteristics were quantified by determining the swelling ratio (g/g) using Equation (7). In this calculation, W_t_ is the mass of the swollen hydrogel and dialysis membrane bag, W0 is the initial dry weight of the hydrogel, and Wc is the weight of the hydrated bag. Furthermore, the normalized swelling parameter (Q_t_) was used to describe the amount of liquid absorbed relative to the original hydrogel mass at a given time (t). This parameter, along with equilibrium swelling, was determined using Equation (8).

(8) $$Q_{t}=\frac{W_{s}-W_{d}}{W_{d}}=\frac{W_{t}}{W_{d}}$$

The swelling behavior was quantified by measuring the hydrogel weight at time t (Ws), its original dry weight (Wd), and the corresponding water uptake (Wt), which was calculated as the difference between Ws and Wd. The equilibrium swelling degree Q_e_ is defined as the ratio of the absorbed medium/buffer to the hydrogel’s dry weight at t = 0 and can be evaluated using Equation (9):

(9) $$Q_{e}=\frac{W_{\infty }-W_{d}}{W_{d}}=\frac{W_{e}}{W_{d}}$$

W∞ represents the mass of the hydrogel after swelling has stabilized at time t∞, W_d_ is the hydrogel’s dry weight at the start (t = 0), and this indicates the water uptake at the equilibrium level. All swelling experiments were performed in triplicate, and the data are presented as mean ± standard deviation.

### 2.14. Hydrogel Swelling Behavior in Saline Media

Hydrogel samples in dialysis membrane bags were immersed in NaCl and KCl solutions ranging from 0.1 to 2.0 M at 25 °C to examine their swelling behavior. The equilibrium swelling after 24 h was measured using Equation (7).

### 2.15. Thermal Influence on Swelling of Hydrogel

To evaluate thermoresponsive swelling, pre-weighed hydrogel samples in dialysis membrane bags were immersed in deionized water at 20, 30, 40, and 50 °C for 24 h. Swollen weights were recorded periodically, and swelling indices were calculated.

### 2.16. Response of Hydrogel to External Stimuli: Swelling and Deswelling Behavior

The swelling and deswelling behavior of HPC hydrogel was evaluated using a gravimetric method. Initially, pre-weighed hydrogel samples were immersed in deionized water for one hour to allow swelling. The samples were then transferred to pure ethanol for one hour to observe deswelling through weight reduction. Further tests were performed to determine the hydrogel’s responsiveness in buffer solutions at pH 7.4 and 1.2. The hydrogel underwent alternating swelling and deswelling phases: first, it was immersed in a pH 7.4 buffer for one hour and then exposed to a pH 1.2 buffer for one hour. The swelling–deswelling cycles were also conducted in parallel with deionized water and 0.9% aqueous NaCl solution. All experiments were performed in triplicate to minimize inherent variability and ensure statistical reliability, with each cycle repeated four times.

### 2.17. Swelling Kinetics

The swelling kinetics of the hydrogel were analyzed using the normalized swelling parameter (Q_t_) and its equilibrium value (Q_e_). A second-order kinetic model was applied to describe the swelling process, as expressed in Equation (10):

(10) $$\frac{t}{Q_{t}}=\frac{1}{KQ_{e}^{2}}\;+\frac{t}{Q_{e}}$$

A plot of $\frac{t}{Qt}$ versus t yields a straight line, with slope 1/Q_e_ and intercept 1/kQ_e_^2^ consistent with pseudo-second-order kinetics.

### 2.18. Water Solubility of Hydrogel

To evaluate the solubility (S, expressed as a percentage) of the hydrogels during their swelling period in water, samples were weighed (in grams) after each drying cycle conducted at 30 °C for 24 h. The solubility of the hydrogel was calculated using Equation (11):

(11) $$S=\left[\frac{Initial\;dry\;weight}{Initial\;dry\;weight-Final\;dry\;weight}\right]\times 100$$

### 2.19. Preparation of Tablets

Diacerein, ciprofloxacin HCl, and febuxostat were used as representative pharmaceutical compounds to evaluate the potential of HPC-based hydrogels HPC-NaCMC-CA (AS1), HPC-CA (AS2), and NaCMC-CA (AS3), respectively. All pharmaceutical tablets were produced by direct compression. Diacerein (50 mg) was mixed with HPC-NaCMC-CA hydrogel (250 mg) in a mortar to form the AS1 formulation, followed by the addition of magnesium stearate (20 mg) as a lubricant. The tablets were then compressed using a single-punch tablet press with a 9 mm flat-faced punch to achieve a hardness of 5–7 kg/cm^2^. Compression pressure was adjusted to obtain tablets within the desired hardness range. For the AS2 formulation, ciprofloxacin HCl (250 mg) was blended with HPC-CA hydrogel (300 mg) and formed into a moist mass using a 5% aqueous tragacanthin solution (*w*/*v*). The damp mass was passed through a No. 12 sieve to obtain granules, which were dried at 60 °C for 3 h, oiled with magnesium stearate (30 mg), and compressed using a 12 mm flat-faced punch to achieve a hardness of 7–9 kg/cm^2^. Similarly, for the AS3 formulation, febuxostat (80 mg) was combined with NaCMC-CA hydrogel (200 mg) and granulated using the same method. The granules were lubricated with 10 mg of magnesium stearate and compressed to a hardness of 7–9 kg/cm^2^. These three formulations are AS1, AS2, and AS3, respectively (Table 2).

### 2.20. Swelling Studies of HPC Hydrogel-Containing Tablets

The swelling capacity of tablets from each formulation was evaluated in distilled water, and the extent of expansion was measured using Equation (8).

### 2.21. Drug Release Behavior and Mechanistic Study Under In Vitro Conditions

HPC hydrogel tablets were placed in USP Dissolution Apparatus II containing phosphate buffer (pH 6.8), maintained at 37 ± 0.5 °C to study drug release. The paddle rotated at a constant speed of fifty rpm throughout the test. At predetermined intervals, 5 mL was withdrawn and analyzed spectrophotometrically at 254 nm for HPC-NaCMC-CA hydrogels, 276 nm for HPC-CA hydrogels, and 314.5 nm for NaCMC-CA hydrogels. At each sampling interval, the same volume of fresh phosphate buffer (pH 6.8) was added to the dissolution medium to maintain sink conditions throughout the study. Diffusion is the dominant mechanism of drug release from swellable polymer matrices, but several other mechanisms may also play a role. This phenomenon can be quantified using the power-law Equation (12) [16].

(12) $$\frac{M_{t}}{M_{\infty }}=K_{p}t^{n}$$

This equation defines Mt/M∞ as a function of time t, where k_p_ is the power-law rate constant and n is the diffusion exponent, which identifies the release mechanism. Determining the type of drug transport is based on the value of n; when n = 0.45, drug release occurs predominantly via Fickian diffusion, governed by molecular diffusion. However, values of n in the range 0.45–0.89 indicate non-Fickian (anomalous) drug diffusion, which signifies that release is influenced by both polymers swelling and diffusion mechanisms.

### 2.22. Drug Content Evaluation

The hydrogels were loaded with the drug using the absorption technique. Dried hydrogel samples were placed in 100 mL of a 1% *w*/*v* drug solution prepared with a 50:50 mixture of phosphate buffer and methanol. The hydrogels were allowed to swell to equilibrium, after which they were removed and dried in an oven at 40 °C until a constant weight was achieved. Drug loading was measured by extracting the drug with the same methanol/phosphate buffer mixture used during loading. The extraction process was repeated regularly with freshly prepared solutions until no detectable drug remained. All extracted samples were analyzed for drug content, and a calibration curve was constructed using serial dilutions to determine the drug concentration spectrophotometrically at 205 nm (λmax). The amount of drug loaded was calculated as (13):

(13) $$Amount\;of\;drug=W_{D}-W_{d}$$

In this context, W_D_ denotes the weight of the dried hydrogel after immersion in the drug solution, and W_d_ refers to its initial weight before immersion.

### 2.23. Evaluation of the Release of a Drug from Hydrogel

Dissolution of the drug from the hydrogel was determined using a dissolution protocol involving 0.1 M USP phosphate buffer solutions at pH 1.2 and 7.4. The hydrogel matrix, including the active agent in its dry form, was interspersed into 500 mL of buffer medium at room temperature and agitated using a paddle-type stirrer at 50 revolutions per minute. Sampling trials were performed at specified time intervals and analyzed for drug release using UV spectrophotometry at the compound’s peak absorbance (λmax) of 205 nm.

### 2.24. Kinetics of Release of Drugs

Various models were used to evaluate and fit the drug release mechanism as described below:

### 2.25. Zero-Order Kinetics of Drug Release

In this model, the drug delivery systems in which the release rate is concentration independent and formulated as:

(14) $$Q_{t}=K_{0}t$$

The amount of drug released at time t is given by Qt in the zero-order kinetic model, with K_0_ as the proportionality factor.

### 2.26. Evaluation Using a First-Order Kinetic Model

Drug release that depends on the concentration of the drug is typically described by first-order kinetics, which can be expressed as follows:

(15) $$LogQ=LogQ_{0}-\left(\frac{K_{1}t}{2.303}\right)$$

In this model, Q_0_ represents the initial amount of drug present, while Q denotes the amount remaining at time t. The parameter K_1_ corresponds to the first-order rate constant governing the drug release process [17,18].

### 2.27. Higuchi Model

A simpler equation for the Higuchi model is as follows:

(16) $$Q_{t}=K_{H}t^{1/2}$$

In this context, Q_t_ represents the drug release over time t, whereas KH is referred to as the Higuchi rate constant, which defines the amount of drug diffusion associated with the matrix [19].

### 2.28. Korsmeyer–Peppas Drug Release Model

To overcome this complexity in release kinetics, a semi-empirical model called the Korsmeyer–Peppas equation has provided a versatile mathematical framework to include classical Fickian diffusion as well as non-Fickian contributions such as polymer relaxation

(17) $$M_{t}/M_{\infty }=Kt^{n}$$

Here, Kt^n^ is the kinetic constant that accounts for the carrier’s geometry and structural features, Mt/M∞ is the drug content released at time t, and n is the diffusion exponent describing the mechanism of release. An n value of approximately 0.45 indicates Fickian diffusion, in which the release is mainly mediated by molecular diffusion. A value of n equal to 1 corresponds to Case II transport, dominated by polymer relaxation and swelling. Values of n between 0.45 and 1 indicate non-Fickian (or anomalous) or super Case II transport, in which diffusion and polymer relaxation contribute to the release process [20,21].

### 2.29. Hixson–Crowell Model

Equation (18), which represents the proportion of drug accumulated in the dissolution agent at time t, was used to explain the dissolution and release processes.

(18) Q013−Qt13 =  −KHCt

Within this model, Q_0_ denotes the initial drug content of AS-1, Qt denotes the release amount at time t, and KHC denotes the kinetic constant for the Hixson–Crowell equation [22].

The Model Selection Criterion (MSC), an adapted version of the Akaike Information Criterion, was utilized to identify the most suitable kinetic model for describing drug release (Equation (19)) [23]. The kinetic model with the highest MSC score is considered the best fit.

(19) $$MSC=\ln\left(\frac{{\sum_{i=1}^{n}w_{\dot{i}}(Y_{{obs}_{i}}-\;{\bar{Y}}_{obs})}^{2}}{{\sum_{i=1}^{n}w_{\dot{i}}(Y_{{obs}_{i}}-\;Y_{{cal}_{i}})}^{2}}\right)-\frac{2p}{n}$$

In this equation, MSC denotes the Model Selection Criterion, n is the total number of experimental data points, and wi denotes the weighting factor for each observation. Y_obsi_ refers to the experimentally observed value at time i, whereas Y_obs_ is the average of all observed values. Y_cali_ corresponds to the value predicted by the kinetic model for the ith data point. The parameter *p* represents the number of adjustable variables (model parameters).

## 3. Results

### 3.1. HPC Hydrogel

The characteristics of finely milled HPC hydrogel powder, sieved through a No. 60 mesh, were assessed. The evaluation included measurements of particle size, moisture content, and flow properties, including the angle of repose. Bulk and tapped densities were determined alongside swelling capacity, while compressibility and flow indices were evaluated using Carr’s index and the Hausner ratio. The centrifuge retention capacity was also measured to further characterize the material’s properties (Table 3).

### 3.2. Morphological Examination with Scanning Electron Microscopy

The morphological and structural details of HPC-based hydrogels were characterized using SEM imaging. SEM revealed a loose 3D network structure with pore sizes ranging from tens to hundreds of micrometers. The pore sizes of the HPC hydrogels were clearly smaller when combined with HPC-Na CMC-C (Figure 1). Although SEM analysis confirmed the presence of a porous interconnected surface morphology, detailed cross-sectional imaging across the membrane thickness was not performed in the present study. Therefore, a direct correlation between internal pore distribution and swelling behavior requires further investigation. This likely occurred because adding Na CMC reduced the gel matrices’ tendency to expand, thus decreasing the pore sizes.

### 3.3. Weight-Loss Profiling by Thermogravimetric Hydrogel Analysis (TGA)

**Figure 2:**
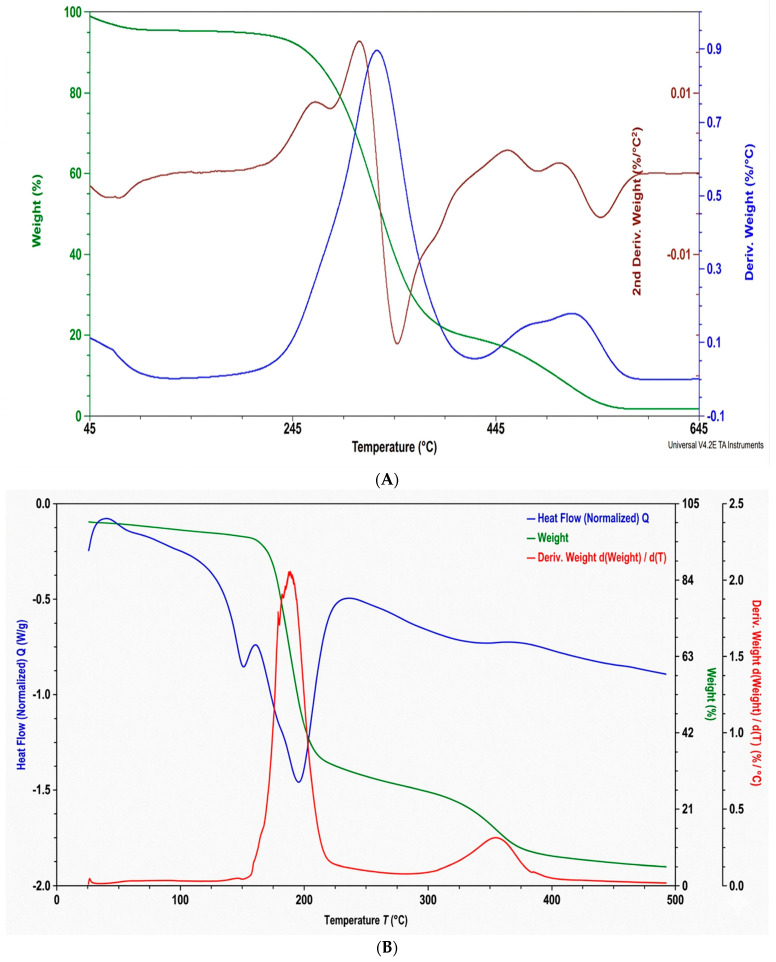

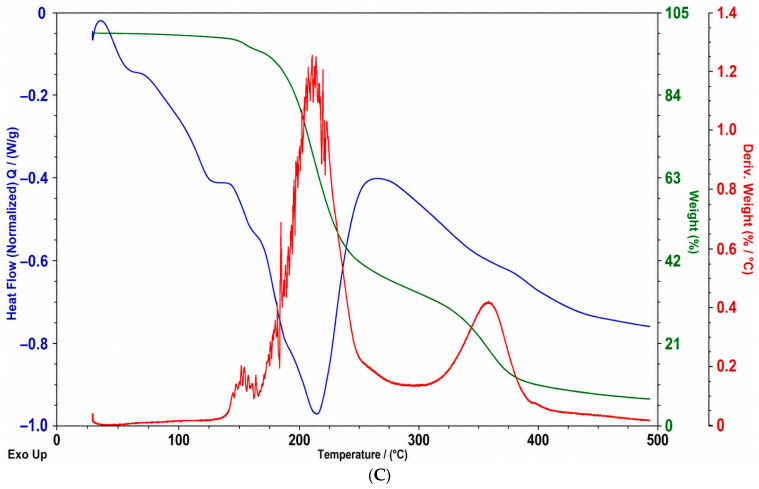
Thermograms of HPC and modified hydrogels: (**A**) HPC; (**B**) HPC-CC; (**C**) HPC-C, showing differences in thermal behavior.

TGA is a technique used to measure the temperatures at which a material undergoes thermal degradation, with mass loss affected by heating duration and temperature. (Figure 2A–C). TGA helps evaluate a material’s stability at high temperatures, whether in the presence or absence of air, and determine the optimal temperature range to enhance its porosity [24]. This complementary method offers insights into the composition and changes in the thermal stability of the samples. The thermal stability of the CA- and NaCMC-based HPC hydrogels was assessed via TGA. Thermal decomposition profiles are presented as disintegration temperatures (T_i_, T_m,_ and T_f_) for each degradation step (Table 4). HPC exhibited single-step degradation, with Ti, Tm, and Tf values of 244, 328, and 396 °C, respectively. Additionally, HPC-CC showed degradation with T_i_, T_m,_ and T_f_ values of 158, 189, and 249 °C, respectively. Conversely, HPC-C displayed T_i_, T_m_, and Tf values of 145, 210, and 295 °C, respectively. The CC exhibited single-step degradation, with Ti, Tm, and Tf values of 136, 177, and 247 °C, respectively.

The Friedman, Chang, and Broido models were also employed to determine the thermal degradation kinetics of HPC, HPC-CC, HPC-C, and CC.

### 3.4. Kinetics of Swelling

To effectively utilize hydrogels in formulations, it is essential to increase both their swelling capacity and rate of swelling. The swelling kinetics of hydrogels are heavily influenced by the inherent swelling capacity, the surrounding pH, and the particle size distribution [25]. Recognizing the critical importance of hydrogels, the swelling kinetics of HPC were examined. The data suggested diffusion-associated swelling behavior of the hydrogel system.

### 3.5. HPC Hydrogel Swelling in Response to Saline Environment

**Figure 3:**
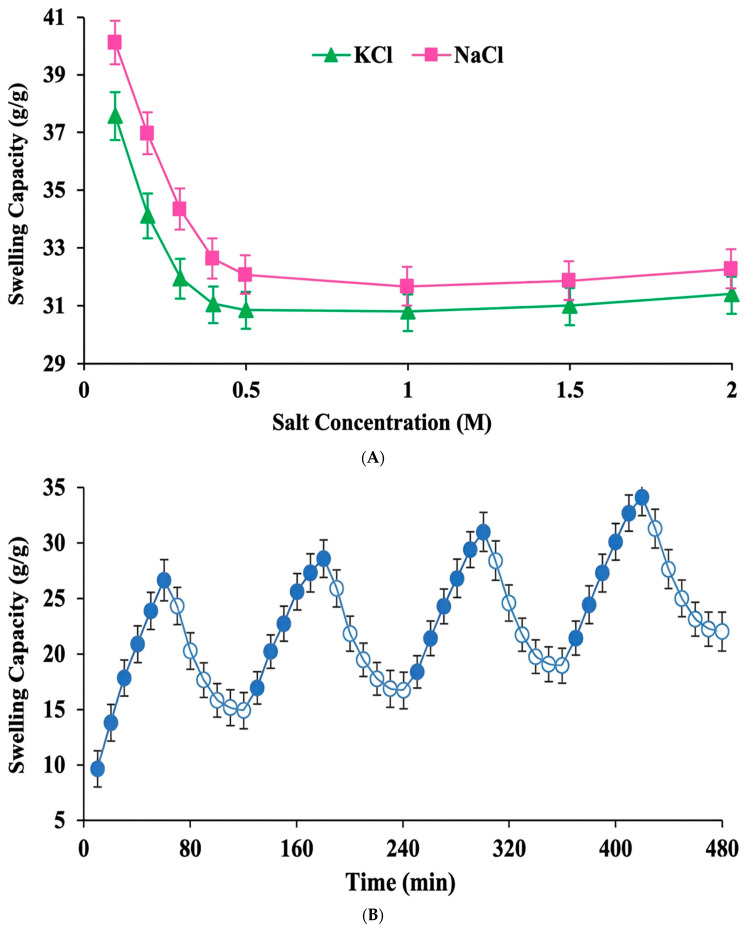

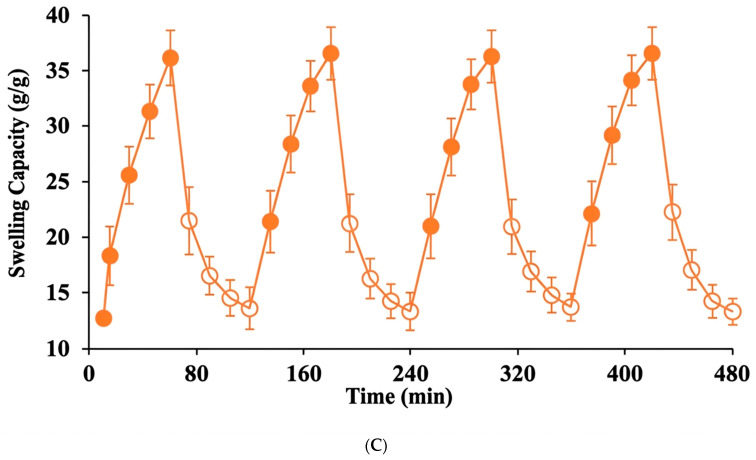
(**A**) Equilibrium swelling of HPC hydrogel after 24 h in NaCl and KCl solutions. (**B**) Reversible swelling behavior of HPC hydrogel in distilled water and saline. (**C**) Reversible swelling behavior of HPC hydrogel in distilled water and ethanol.

The extent of naturally generated polysaccharide expansion depends on the presence of hydrophilic groups in hydrogels or hydrophobic groups, as well as on the extent of cross-linking and the structure’s flexibility. Additionally, the expansion of hydrogels is significantly influenced by changes in salt concentration within the expansion medium [11]. As a result, the effect of increasing ionic concentrations of NaCl and KCl on the expansion percentage of HPC hydrogel was examined. The equilibrium expansion sharply decreases when the salt concentration in the aqueous solution rises from 0.1 to 0.5 M. This reduction in expansion may be caused by the electric-charge-screening effect of abundant cations interacting with ionizable groups present in the modified hydrogel formulations, which leads to weaker anion-anion repulsive forces [26]. The repulsive interactions reduce the osmotic pressure gradient between the polymer and the external solution, leading to polymer dehydration in the aqueous environment [27]. Furthermore, the hydrogel’s increased affinity for K+ ions also results in decreased swelling in the KCl solution (Figure 3A). This reduction in swelling is attributed to ionic screening effects within the modified hydrogel formulations.

### 3.6. Reversible Swelling–Deswelling Characteristics of HPC Hydrogel in Water and Saline Solutions

HPC hydrogel powder was tested for its ability to swell and shrink in pure water and in a 0.9% sodium chloride solution. The hydrogel swelled rapidly in deionized water, whereas exposure to the 0.9% NaCl solution caused it to shrink. This behavior can be attributed to the pressure difference generated by osmotic forces between the polymer network of the HPC hydrogel and the surrounding solution. When swollen HPC hydrogel in powder form encounters 0.7% NaCl, it experiences a reduction in osmotic pressure due to the presence of Na+ ions in the solution, which causes water molecules to flow out of the gel and shrink the hydrogel. Similarly, when the dehydrated HPC hydrogel is introduced into distilled water, Na+ cations are washed away, and osmotic pressure recovers, leading to the HPC hydrogel powder swelling again (Figure 3B).

### 3.7. On–Off Swelling Behavior of HPC Hydrogel in Aqueous and Ethanol Media

Powdered HPC hydrogel was tested for swelling and deswelling in water and ethanol to evaluate its stimulus-responsive behavior. It was observed that, after swelling, the hydrogel rapidly deswelled in ethanol. This rapid deswelling is caused by ethanol replacing water molecules, accelerating the hydrogel’s dehydration. The swelling–deswelling cycle in water and ethanol was repeated four times (Figure 3C). This solvent-induced deswelling significantly reduces hydrogel swelling and water uptake.

### 3.8. Swelling at Different Temperatures

**Figure 4:**
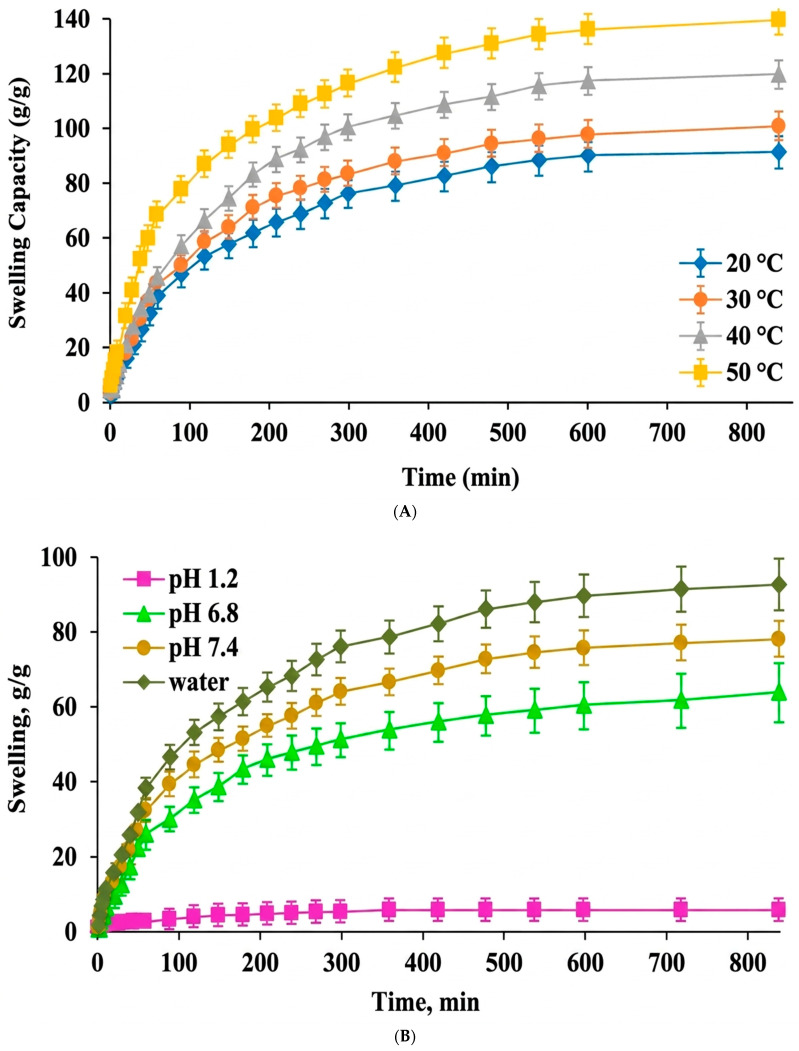

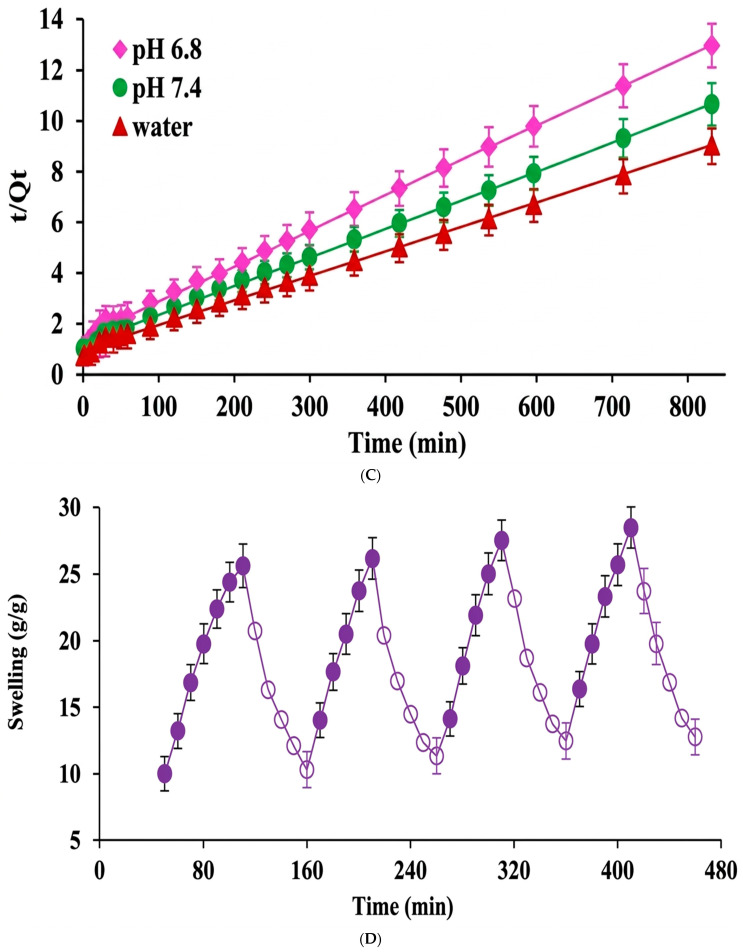
(**A**) Effect of temperature on the equilibrium swelling ratio of the HPC-based hydrogel, indicating thermal responsiveness. (**B**) Time-dependent swelling kinetics of the HPC-based hydrogel in distilled water and under physiological pH conditions (6.8 and 7.4). (**C**) Swelling capacity of the HPC-based hydrogel in distilled water and buffer solutions at pH 1.2, 4.5, 6.8, and 7.4, demonstrating pH-dependent behavior. (**D**) Cyclic swelling and deswelling responses of the HPC-based hydrogel at pH 7.4 and 1.2, illustrating reversible pH-sensitive behavior.

The temperature-dependent swelling behavior of the pre-weighed hydrogel was investigated by placing it in dialysis membrane bags in deionized water at 20, 30, 40, and 50 °C for 24 h (Figure 4A). The hydrogel’s weight was periodically measured, and the swelling index was subsequently determined.

### 3.9. Influence of pH on the Swelling Performance of HPC Hydrogels in Distilled Water and Buffer Solutions

The swelling behavior of the HPC hydrogel was studied at pH values mimicking different regions of the gastrointestinal tract. Test media included a simulated gastric solution (pH 1.2), a mildly acidic buffer representing the small intestine (pH 6.8), and a basic buffer corresponding to the large intestine (pH 7.4), along with distilled water for comparison (Figure 4B). The modified HPC-based hydrogel formulations swelled quickly in distilled water at pH 6.8 and pH 7.4, whereas swelling was significantly reduced at pH 1.2. This suggests reduced swelling under acidic conditions and enhanced swelling under neutral conditions. Further investigation showed the highest expansion in distilled water and lower swelling at pH 7.4 and 6.8 (Figure 4C). The slight decrease in swelling at pH 7.4 compared to distilled water is probably due to Na^+^ ions shielding ionizable carboxylate groups contributed mainly by NaCMC and citric acid components, which reduces electrostatic repulsion between anionic sites [28].

### 3.10. pH-Dependent Swelling–Deswelling Characteristics of HPC Hydrogel

When exposed to a pH 7.4 solution, the HPC hydrogel expanded quickly, and subsequent transfer to pH 1.2 caused rapid shrinkage, confirming its pH-sensitive on–off switching. This behavior is attributed mainly to ionizable carboxyl-containing groups introduced through NaCMC and citric acid within the modified hydrogel formulations. The carboxyl-containing groups in the modified hydrogel formulations remain protonated at pH 1.2 and form intra- and intermolecular hydrogen bonds with the polysaccharide network, leading to hydrogel dehydration. At pH 7.4, ionization of carboxyl-containing groups within the modified hydrogel formulations generates electrostatic repulsion between identical charges, thereby enhancing swelling. Disruption of hydrogen bonds due to repulsion causes the hydrogel chains to extend, promoting rapid swelling. Alternating swelling–shrinking cycles of HPC hydrogel powder occur under physiological (pH 7.4) and gastric (pH 1.2) conditions (Figure 4D). Even after multiple swelling–deswelling cycles, the HPC hydrogel continues to exhibit pH-responsive properties, demonstrating its reversible pH sensitivity.

### 3.11. The Investigation of Tablet Swelling

The assessment of the swelling ability of HPC hydrogel tablets was performed in distilled water. Polymer swelling begins when water molecules interact with the tablets. The hydrophilic groups within the polymer network attract water. Ionizable carboxyl-containing groups contributed mainly by NaCMC and citric acid tend to dissociate into ions allowing water molecules to penetrate deeper into the polymer framework due to electrostatic repulsions among these ions [29]. This loosening of the 3D polymer framework allows more water to seep in, thereby increasing hydrogel swelling. The swelling rate depended on the HPC hydrogel content. Dissolution and dispersion of tablets containing a higher percentage of HPC hydrogel were found to be greater than those containing a lower amount of HPC hydrogel. The overall scale of swelling was unaffected by the drug dosage included. The tablets were placed in distilled water for visual assessment, and images were captured at regular intervals.

### 3.12. Drug Release

Drug release was measured using a dissolution method with 0.1 M USP-buffered phosphate mixtures at pH 7.4 and 1.2. In vitro release testing involved immersing the drug-incorporated hydrogel in 500 mL of buffered solution at room temperature, with continuous paddle stirring at 50 rpm. Samples taken at specific intervals were analyzed for drug concentration using UV spectrophotometry at 205 nm (λmax).

### 3.13. Assessment of Drug Release Under In Vitro Conditions

**Figure 5:**
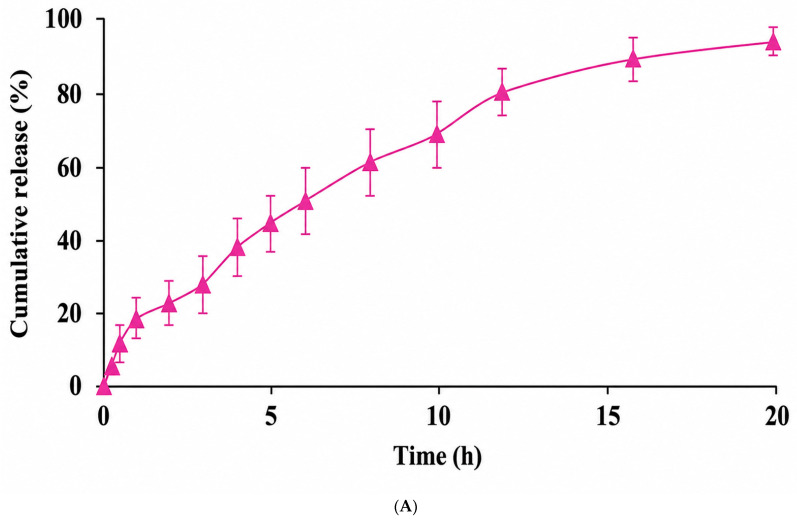

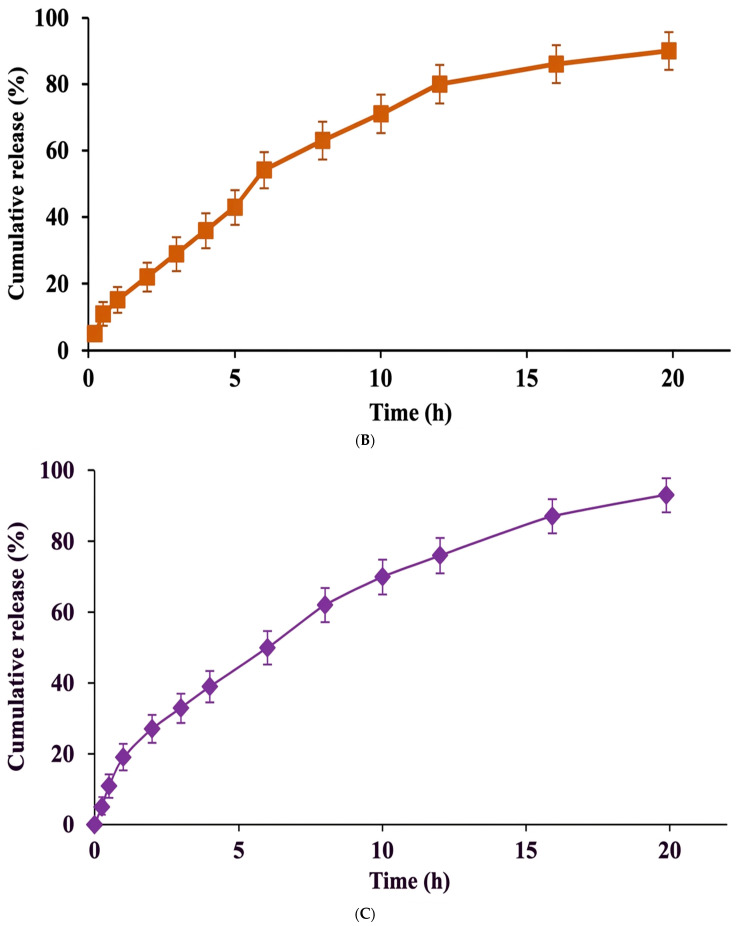
Drug release profiles of HPC-based hydrogel formulations at pH 6.8: (**A**) AS1; (**B**) AS2; (**C**) AS3, illustrating comparative release behavior under simulated physiological conditions.

Key factors influencing drug release from polymeric delivery systems include the hydrogel’s swelling behavior, the drug’s solubility in the release medium and the polymer matrix, and drug interactions [30]. The drug release from HPC-NACMC-CA hydrogel-based tablets labeled AS1 was tested at pH 6.8. 93% of the drug was released after 16 h, indicating delayed-release behavior of AS1 (Figure 5A). Ciprofloxacin release from HPC-CA hydrogel-based tablets labeled AS2 was also tested at pH 6.8. It was observed that 90% of the drug was released after 20 h, demonstrating delayed release of AS2 (Figure 5B). The drug release from NACMC-CA hydrogel-based tablets labeled AS3 was tested at the same pH. 90.7% of the drug was released after 20 h, indicating sustained release behavior of AS3 (Figure 5C). These results suggest that AS1, AS2, and AS3 are capable of sustaining drug release at pH 6.8, which corresponds to the pH of the small intestine. These findings suggest potential applicability for delayed, sustained, or prolonged drug release, as well as targeted delivery to the small intestine. These observations suggest that drug release may be influenced by diffusion through the swollen hydrogel matrix.

### 3.14. Drug Release Kinetics and Its Mechanism

The model with an R^2^ value closest to 1 is considered the most suitable. Using this metric, the Korsmeyer–Peppas model was found to best represent the drug release profiles of AS1, AS2, and AS3. Similarly, the model with the highest MSC value is considered the most appropriate, further supporting the conclusion that the Korsmeyer–Peppas model best describes drug release from AS1, AS2, and AS3. Various kinetic models were employed to analyze drug release (Table 5).

Although the Higuchi model showed good agreement with the release data for AS3, the Korsmeyer–Peppas model provided the best overall fit based on both R^2^ and MSC values. Similarly, the MSC analysis also supported the Korsmeyer–Peppas model as the most appropriate kinetic model for describing drug release from AS3. The Korsmeyer–Peppas model indicated that drug release from the hydrogel follows a non-Fickian diffusion mechanism involving swelling and diffusion, with n values of 0.667 for AS1, 0.796 for AS2, and 0.556 for AS3.

## 4. Discussion

Details of cross-linked HPC hydrogels’ swelling performance, responsiveness to environmental changes, and release kinetics were investigated to confirm their suitability for controlled drug delivery. The deionized water, as well as the pH 6.8 and 7.4 swelling parts, showed large expansion, whereas acidic conditions (pH 1.2) resulted in apparent inhibition of swelling. Such a pH-controllable swelling behavior is consistent with previous studies on pH-sensitive hydrogels, in which osmotic swelling pressure is driven by ionizable groups contributed mainly by NaCMC and citric acid within the modified HPC-based formulations (the protonation and deprotonation of COOH into COO^−^ functional groups) determines polymer chain repulsion [3,28]. The notable shrinkage observed at pH 1.2 (acidic) may be attributed to the protonation of carboxyl-containing groups in the modified hydrogel formulations, leading to hydrogen bonding and thus assisting in the contraction of the polymer chain [25,31]. Phase transition behavior as a function of pH further enables site-specific drug delivery by restricting swelling in acidic gastric fluid and promoting greater expansion in neutral to slightly basic intestinal media, thereby optimizing release timing. These findings support the interpretation of the modified HPC-based hydrogel system as a stimuli-responsive material in which environmental conditions influence swelling-associated diffusion behavior. The swelling process followed a pseudo-second-order kinetic model, in which the rate depends on the extent of swelling potential the hydrogel can attain and the dominant environmental conditions, consistent with previously reported swelling behavior of hydrophilic polymeric hydrogel systems [9,10]. Also, the swelling rate was temperature-dependent; higher temperatures (20–50 °C) led to greater swelling, corroborating the thermoresponsive behavior of HPC reported [7,32]. These interactions have been demonstrated by reversible swelling–deswelling cycles when the HPC hydrogel was exposed to various solvents (water and ethanol) and ionic solutions (deionized water and saline), demonstrating the stimuli-responsive behavior of the hydrogel system. This type of reversible swelling–deswelling switching is mainly ascribed to variations in osmotic pressure and polymer-solvent interactions, consistent with previous literature reporting similar behavior in “smart” hydrogels [4,9]. Rapid deswelling in ethanol further indicates the hydrogel’s responsiveness to solvent polarity and hydrogen-bonding capacity [11]. Such responsiveness can be leveraged for the controlled delivery of drugs, in which external stimuli govern drug release. The reduction in swelling behavior of hydrogels in saline (NaCl, KCl) solutions was attributed to ionic screening, which decreased the net electrostatic repulsion within the hydrogel network, leading to polymer shrinkage and dehydration [26,27]. Previous studies have also shown cation-specific interactions with polysaccharide hydrogels, which support our findings, as KCl binds more strongly and swells less than NaCl [11]. These findings imply that HPC hydrogels can be tailored for applications in media with different ionic strengths, including physiologic fluids. Thermogravimetric analysis indicated that HPC hydrogels have very good thermal stability (decomposition began at >140 °C and lost most of its weight at about 240 °C), consistent with previous reports on cellulose derivatives [6,24]. This thermal stability ensures the structural integrity of HPC-based drug delivery systems during manufacturing and storage. The swelling behavior and porous microstructure of these hydrogels also enable a high capacity for drug loading via absorption, corroborating previous reports on the hostability of hydroxypropyl cellulose for hydrophilic drugs [7,8]. The in vitro study showed sustained drug release at pH 6.8, and the drug release kinetics were best described by the Korsmeyer–Peppas model for AS1, AS2, and AS3 formulations, suggesting a non-Fickian drug release mechanism involving both diffusion and polymer relaxation processes [20]. The Korsmeyer–Peppas model indicated that drug release was governed by a combination of diffusion and polymer relaxation mechanisms. The Korsmeyer–Peppas model suggests a non-Fickian release mechanism in which drug diffusion and polymer network relaxation occur concurrently [20], a behavior commonly observed in hydrogel-based drug delivery systems [16]. The observed sustained drug release over 16–20 h at intestinal pH highlights the HPC hydrogel’s suitability for colon-targeted and prolonged-release oral formulations.

## 5. Conclusions

The modified HPC-based hydrogel formulations containing NaCMC and citric acid showed intense swelling in water and at various gastrointestinal pH levels. The rapid shrinkage observed at pH 1.2 may contribute to reduced drug release under acidic gastric conditions. On the other hand, owing to a significant degree of swelling at pH 6.8 and 7.4, it has potential for sustained drug delivery to the colon. It also exhibited highly reversible swelling–deswelling cycles upon alternate exposure to water and saline, water and ethanol, or acidic and basic buffers, suggesting that the modified HPC-based hydrogel system can behave as a stimuli-responsive hydrogel system with potential for controlled diffusion-mediated drug release applications. These modified HPC-based hydrogels, known for their excellent swelling ability, responsive switching, and highly porous structure, hold substantial promise for future pharmaceutical and biomedical applications. Future work may include a quantitative evaluation of membrane permeability coefficients to characterize transport performance further.

## Figures and Tables

**Table 1:** Composition and preparation conditions of HPC-based hydrogel formulations.

Hydrogel Formulation	HPC (g)	NaCMC (g)	Citric Acid (g)	Water Volume (mL)	Total Polymer Concentration (% *w*/*v*)
HPC-NaCMC-CA	3.0	9.0	15.0	100	12
HPC-CA	4.0	—	8.0	100	4
NaCMC-CA	—	9.0	15.0	100	9

**Table 2:** Composition of Formulation for Investigating HPC Hydrogel to create an oral drug dose system.

Formulation Concentrations (mg/Tablet)	AS1	AS2	AS3
Synthesized hydrogel	250	300	200
Febuxostat	0	0	80
Ciprofloxacin HCl	0	250	0
Diacerein	50	0	0
Magnesium stearate	20	30	10

**Table 3:** Characteristics of HPC hydrogel powder.

Characteristics	HPC Hydrogel
Residual water content (%)	0.6 ± 0.02
Average particle diameter (μm)	≈280
Repose Angle	59.67 ± 0.36
Untapped bulk density (g/mL)	0.63 ± 0.05
Tapped/Untapped bulk density (g/mL)	0.76 ± 0.07
Carr’s index (%)	51.05 ± 2.87
Density ratio (Hausner factor)	1.80 ± 0.05
Fluid retention after centrifugation (%)	87.26 ± 1.63
Swelling capacity after 24 h (g/g)	32.33 ± 1.18
Gel fraction (%)	37.23 ± 0.59

**Table 4:** TGA-derived data for HPC-based and related composites, indicating thermal.

Hydrogel Sample	Steps	T_i_ (°C)	T_m_ (°C)	T_f_ (°C)	Weight Loss Percentage at T_f_	Char Yield Weight Percentage
HPC	I	244	328	396	71.47	1.74 at 600 °C
HPC-CC	I	158	189	249	65.45	5.26 at 500 °C
HPC-C	I	145	210	295	63.84	6.74 at 500 °C
CC	I	136	177	247	44.17	17.52 at 500 °C

**Table 5:** Drug release kinetics model at pH 6.8.

Model	Parameter	F1	F2	F3
Zero order	*R* ^2^	0.9195	0.9484	0.8314
	K_0_	6.682	36.460	5.240
	MSC	2.3534	2.6783	1.5987
First order	*R* ^2^	0.9843	0.9464	0.9759
	K_1_	0.122	0.707	0.109
	MSC	3.9867	2.6399	3.5427
Higuchi	*R* ^2^	0.9634	0.8850	0.9922
	K_H_	21.233	52.708	19.582
	MSC	3.1416	1.8768	4.6755
Korsmeyer–Peppas	*R* ^2^	0.9969	0.9767	0.9974
	K_KP_	14.783	43.143	17.125
	*N*	0.667	0.796	0.556
	MSC	5.4530	3.1861	5.5948
Hixson–Crowell	*R* ^2^	0.9864	0.9787	0.9568
	K_HC_	0.034	0.196	0.030
	MSC	4.1344	3.5630	2.9593

## Data Availability

The raw data supporting the conclusions of this article will be made available by the authors, without undue reservation.
